# Effects of Laparoscopic Sleeve Gastrectomy on Vascular and Myocardial Recovery: Type 2 Diabetes and Echocardiographic Indices of Diastolic Function

**DOI:** 10.3390/medicina62101896

**Published:** 2026-09-30

**Authors:** Samet Yılmaz, Kayıhan Karaman, Zeki Özsoy, Ataç Çelik

**Affiliations:** 1 Department of Cardiology, Faculty of Medicine, Başkent University, Adana Dr. Turgut Noyan Application and Research Center, Adana 01250, Türkiye; 2 Department of Cardiology, Faculty of Medicine, Tokat Gaziosmanpaşa University, Tokat 60030, Türkiye; kay55han@hotmail.com (K.K.); dretaci@yahoo.com (A.Ç.); 3 Department of General Surgery, Medical Park Hospital, Tokat 60100, Türkiye; zekiserkanozsoy@hotmail.com

**Keywords:** sleeve gastrectomy, bariatric surgery, obesity, type 2 diabetes mellitus, diastolic function, reverse remodelling, carotid intima-media thickness, echocardiography

## Abstract

*Background and Objectives*: Obesity affects both the arterial wall and the myocardium, but after bariatric surgery the two compartments are usually studied in separate cohorts. We measured both in the same patients before and six months after laparoscopic sleeve gastrectomy (LSG), and asked whether type 2 diabetes mellitus (T2DM) is associated with differences in postoperative cardiac changes. *Subjects and Methods*: Fifty consecutive adults with severe obesity scheduled for LSG were enrolled prospectively at a single centre. Thirty completed the six-month protocol; 20 were not reassessed during pandemic-related disruption of elective outpatient follow-up. Carotid intima-media thickness (CIMT), ankle-brachial index (ABI), aortic propagation velocity (APV) and transthoracic echocardiography were obtained at both timepoints by the same operator, blinded to diabetes status and laboratory results, on the same equipment. Left atrial volume index and tricuspid regurgitant velocity were not recorded, so diastolic function was assessed using continuous indices (E/A, mean e′, E/e′) rather than formal categorical grading. *Results*: Body mass index fell from 45.5 ± 6.6 to 32.5 ± 5.8 kg/m^2^ (*p* < 0.001). CIMT decreased (0.80 ± 0.12 to 0.66 ± 0.11 mm, d = −1.86) and APV increased (52.0 ± 10.1 to 67.4 ± 10.8 cm/s, d = 1.99; both *p* < 0.001). Mean ABI did not change (1.07 ± 0.21 to 1.06 ± 0.07, d = −0.08, *p* = 0.679). Interventricular septal and posterior wall thickness and left atrial diameter decreased, and E/A and mean e′ increased (all *p* ≤ 0.012), while E/e′, an estimate of filling pressure, was unchanged (*p* = 0.871). Patients with T2DM (*n* = 18) showed a smaller increase in mean e′ than those without (*n* = 12): Δmean e′ 0.44 ± 1.24 versus 1.71 ± 1.68 cm/s (*p* = 0.025). The T2DM group also lost less weight (ΔBMI −11.9 ± 3.1 versus −14.7 ± 3.0 kg/m^2^, *p* = 0.019); after adjustment for baseline mean e′ and ΔBMI, T2DM remained associated with a smaller increase in mean e′ (β = −1.07, 95% CI −2.10 to −0.03, *p* = 0.044), as did a mixed-effects time-by-diabetes interaction (β = −1.26, *p* = 0.018). No statistically significant difference in vascular changes according to diabetes status was detected. *Conclusions*: Carotid intima-media thickness and aortic propagation velocity improved within six months of LSG, alongside favourable changes in several cardiac structural and diastolic indices, while ABI and E/e′ were unchanged. T2DM was associated with a smaller increase in mean e′ after adjustment for weight loss. The cohort is small, lost 40% of enrolled patients to pandemic-related attrition and had no non-surgical control group, so regression to the mean cannot be separated from the postoperative changes observed and these observations remain hypothesis-generating.

## 1. Introduction

Obesity damages the cardiovascular system on two fronts at once. It accelerates arterial wall thickening and stiffening, and it remodels the myocardium [1]. A 2024 European Society of Cardiology consensus statement treats obesity as a direct cause of this injury rather than as shorthand for the risk factors that accompany it [2,3].

The myocardial consequences are well described. Concentric remodelling, atrial enlargement and impaired relaxation appear long before symptoms do [4,5,6,7], placing patients on the path towards heart failure with preserved ejection fraction [8]. A recent Heart Failure Association statement identifies obesity-related heart failure with preserved ejection fraction as a distinct phenotype that responds to large weight loss [9,10].

Bariatric surgery produces weight loss of a magnitude no other intervention reliably matches [11,12] and is associated with fewer cardiovascular events over the long term [13]. It therefore offers a natural experiment in whether early cardiovascular remodelling is reversible. Recent work has advanced this question in two directions: cardiac reverse remodelling has been shown to correlate only weakly with the magnitude of weight loss [14], and speckle-tracking studies have begun to show that patients with obesity and diabetes show smaller improvements in myocardial mechanics than those with obesity alone [15].

What remains unclear is whether the two compartments change concurrently, and in whom. Vascular and cardiac outcomes after bariatric surgery have almost always been reported in separate cohorts [16,17,18,19], so their relationship within a single group of patients is largely unexamined. Nor is it established whether type 2 diabetes mellitus (T2DM)—which drives myocardial fibrosis and microvascular dysfunction independently of adiposity [20,21]—is associated with smaller postoperative cardiac changes when both compartments are measured together.

We hypothesised that laparoscopic sleeve gastrectomy (LSG) is followed by improvement in measured vascular and cardiac indices, and that T2DM is associated with smaller changes in diastolic indices after adjustment for the degree of weight loss. The objective of this study was to measure vascular and cardiac indices in the same cohort before and six months after LSG and to test these two hypotheses, with the relationship between vascular and diastolic change examined as a secondary, exploratory question.

## 2. Subjects and Methods

### 2.1. Recruitment of Patients

This was a prospective observational study conducted at Tokat Gaziosmanpaşa University Faculty of Medicine, Tokat, Türkiye. Surgery was performed by a single surgical team and all vascular and echocardiographic assessments were carried out in the Department of Cardiology of the same institution. Candidacy for bariatric surgery was established by an endocrinologist and a general surgeon. Eligibility for LSG was based on the standard bariatric surgery criteria applied at the time of enrolment: BMI ≥ 40 kg/m^2^ regardless of comorbidity, or BMI ≥ 35 kg/m^2^ in patients with an obesity-related comorbidity. Patients were enrolled consecutively following approval by the institutional ethics committee; no patient was enrolled before the committee’s decision.

All procedures were performed laparoscopically under general anesthesia by the same surgeon and surgical team. The greater curvature was devascularized using a harmonic scalpel, and the fundus was completely mobilized to the left diaphragmatic crus. Sleeve gastrectomy was performed over a 32–36 Fr bougie using four or five sequential laparoscopic stapler firings according to gastric length, followed by an intraoperative methylene blue leak test. Postoperatively, patients underwent routine clinical monitoring and were managed according to the institution’s standard bariatric postoperative pathway, including progressive dietary advancement and scheduled outpatient follow-up.

Fifty patients met the eligibility criteria and completed baseline assessment. Twenty (40.0%) were not reassessed during pandemic-related disruption of elective outpatient follow-up. Individual reasons for loss to follow-up were not systematically recorded. The analysis population was therefore the 30 patients with complete paired vascular and echocardiographic data. Baseline characteristics of completers and non-completers are compared in Supplementary Table S6.

The study was approved by the Clinical Research Ethics Committee of Tokat Gaziosmanpaşa University Faculty of Medicine (project no. 19-KAEK-012; decision of meeting 2019/01 on 8 January 2019, notified 12 February 2019) and conducted in accordance with the Declaration of Helsinki. Written informed consent was obtained from all participants. Reporting follows the STROBE statement.

### 2.2. Inclusion Criteria

Adults with severe obesity accepted for LSG by the institutional bariatric board, able to give written informed consent, and willing to attend the six-month reassessment.

### 2.3. Exclusion Criteria

Patients were excluded at enrolment for coronary artery disease, heart failure, cardiomyopathy, severe valvular disease, myocarditis, peripheral artery disease, complete bundle branch block, atrial fibrillation, permanent pacing, chronic liver disease, malignancy, collagen vascular disease, thyroid dysfunction or chronic renal failure, or if they declined participation. Screening was performed prospectively at the bariatric board and the number of patients excluded under each individual criterion was not recorded separately, which is acknowledged in Section 4.5.

### 2.4. Collection of Data

Height, weight, body mass index (BMI), blood pressure, smoking status and comorbidities were recorded at baseline and at six months. All paired imaging assessments were performed by the same experienced operator, who was blinded to diabetes status and laboratory results at the time of image acquisition and analysis, using the same equipment at both timepoints (Affiniti 70; Koninklijke Philips N.V., Amsterdam, The Netherlands). Blinding to patient identity and study timepoint was not feasible because of the small cohort and the visible postoperative changes in body habitus.

### 2.5. Criteria of Diagnosis

Hypertension was defined as an established diagnosis or the use of antihypertensive medication at enrolment. T2DM was defined as an established diagnosis or treatment with glucose-lowering therapy. Hyperlipidaemia was defined as an established diagnosis or the use of lipid-lowering therapy.

### 2.6. Echocardiography

Transthoracic echocardiography quantified interventricular septal (IVS) and posterior wall (PW) thickness, left atrial (LA) diameter, left ventricular ejection fraction, and diastolic indices (E/A, mean e′, E/e′) according to the 2016 ASE/EACVI recommendations [22]. Mean e′ was calculated as the average of septal and lateral mitral annular early diastolic velocities. Left atrial volume index and tricuspid regurgitant velocity were not recorded, so formal categorical grading of diastolic function was not possible; diastolic findings are therefore reported as directional change in continuous indices and not as diastolic dysfunction grades.

### 2.7. Vascular Assessment

CIMT was measured with a 7.5 MHz linear probe, the patient supine with the head slightly extended. Imaging began at the clavicle and extended cranially to the common carotid bulb. A 1 cm segment within the first 2 cm proximal to the bulb was interrogated bilaterally and three measurements per side averaged [17,18,19].

APV was obtained from the suprasternal window with the probe parallel to aortic flow. The Nyquist limit was set between 30 and 50 cm/s; M-mode recordings were acquired at a sweep speed of 200 mm/s, producing a flame-shaped time–space velocity map. Aliasing velocity was adjusted individually to give a clear velocity slope. APV was the ratio of the distance between the start and end of the slope to time, averaged over at least three measurements, and is reported in cm/s.

ABI was measured at rest with a 10–12 cm cuff placed above the ankle. Dorsalis pedis and posterior tibial systolic pressures were obtained bilaterally with a handheld Doppler. ABI was the highest ankle systolic pressure divided by the highest brachial systolic pressure; the lower limb value was used [23].

### 2.8. Laboratory Data

Venous blood samples were drawn in the morning after an overnight fast of at least eight hours, at baseline and at the six-month visit. Glucose, glycated haemoglobin (HbA1c), creatinine, electrolytes, a complete lipid panel and a full blood count were measured in the institutional central laboratory by standard automated methods.

### 2.9. Statistical Analysis

Normality was assessed with the Kolmogorov–Smirnov test. Normally distributed variables are given as mean ± standard deviation, non-normally distributed variables as median (interquartile range), and categorical variables as n (%). Paired comparisons used the paired *t*-test with the Wilcoxon signed-rank test as confirmation, and the McNemar test for paired categorical data. Cohen’s d was the mean within-patient difference divided by its standard deviation, with 95% confidence intervals from 5000 bias-corrected and accelerated bootstrap resamples of the paired differences (fixed random seed).

The diabetes hypothesis was tested in three ways: subgroup comparison of six-month change scores; multivariable linear regression of Δmean e′ on T2DM status with baseline mean e′ and ΔBMI entered simultaneously, without stepwise selection; and a mixed-effects model with fixed effects for time, diabetes status and their interaction, and a random intercept per patient. The same models were repeated with E/A as the outcome. With 30 observations and three predictors, the sample-to-variable ratio of the multivariable model is at the lower end of what is usually recommended, so parameter estimates should be regarded as unstable and potentially subject to overfitting; the mixed-effects model is reported alongside as a complementary longitudinal analysis, although it remains limited by the same 30 participants.

Comparison of completers with non-completers used Welch’s independent-samples *t*-test for continuous variables and Fisher’s exact test for categorical variables. The secondary vascular–diastolic question was addressed by correlating six-month change scores across domains, each with a leave-one-out sensitivity analysis; these are exploratory and unadjusted for multiplicity. Analyses were performed using IBM SPSS Statistics version 18.0 (IBM Corp., Armonk, NY, USA); Python 3 (SciPy, statsmodels) was additionally used during manuscript preparation for selected supplementary calculations and for verification of the reported analyses. Two-sided *p* < 0.05 was considered significant. Extended detail, sensitivity analyses and the full correlation matrix are in the Supplementary Materials.

## 3. Results

### 3.1. Patients

Thirty patients completed the six-month protocol: 17 women (56.7%) and 13 men (43.3%), mean age 43.8 ± 10.6 years. Eighteen (60.0%) had T2DM and 21 (70.0%) had hypertension at baseline.

Compared with the 20 patients who did not complete follow-up, completers were younger (43.8 ± 10.6 versus 52.4 ± 5.8 years, *p* = 0.001), less often hyperlipidaemic (36.7% versus 75.0%, *p* = 0.010) and had a slightly higher baseline ejection fraction (63.2 ± 2.6% versus 60.5 ± 3.5%, *p* = 0.006). No statistically significant difference was detected in sex, BMI, blood pressure, prevalence of hypertension or T2DM, HbA1c, CIMT, APV, ABI, IVS or PW thickness, LA diameter, E/A, mean e′ or E/e′ (all *p* ≥ 0.057); this descriptive comparison was not powered to establish equivalence (Supplementary Table S6).

### 3.2. Weight Loss and Metabolic Change

BMI fell from 45.5 ± 6.6 to 32.5 ± 5.8 kg/m^2^ (*p* < 0.001). Systolic and diastolic blood pressure, fasting glucose, HbA1c, total cholesterol, low-density lipoprotein cholesterol and triglycerides all decreased, and high-density lipoprotein cholesterol increased [24]. Current smoking fell from 46.7% to 16.7% (Table 1). Hypertension, type 2 diabetes and hyperlipidaemia were defined at baseline by an established diagnosis or the use of the corresponding therapy; because no prespecified remission criteria were applied and medication was not systematically recorded at both timepoints, these comorbidities are reported as baseline characteristics only and their six-month status is not presented. Weight loss was therefore of sufficient magnitude to examine postoperative changes in both compartments.

### 3.3. Vascular and Cardiac Changes

CIMT fell from 0.80 ± 0.12 to 0.66 ± 0.11 mm (d = −1.86) and APV rose from 52.0 ± 10.1 to 67.4 ± 10.8 cm/s (d = 1.99; both *p* < 0.001). Mean ABI did not change (1.07 ± 0.21 to 1.06 ± 0.07, d = −0.08, 95% CI −0.43 to 0.30, *p* = 0.679).

**Figure 1:**
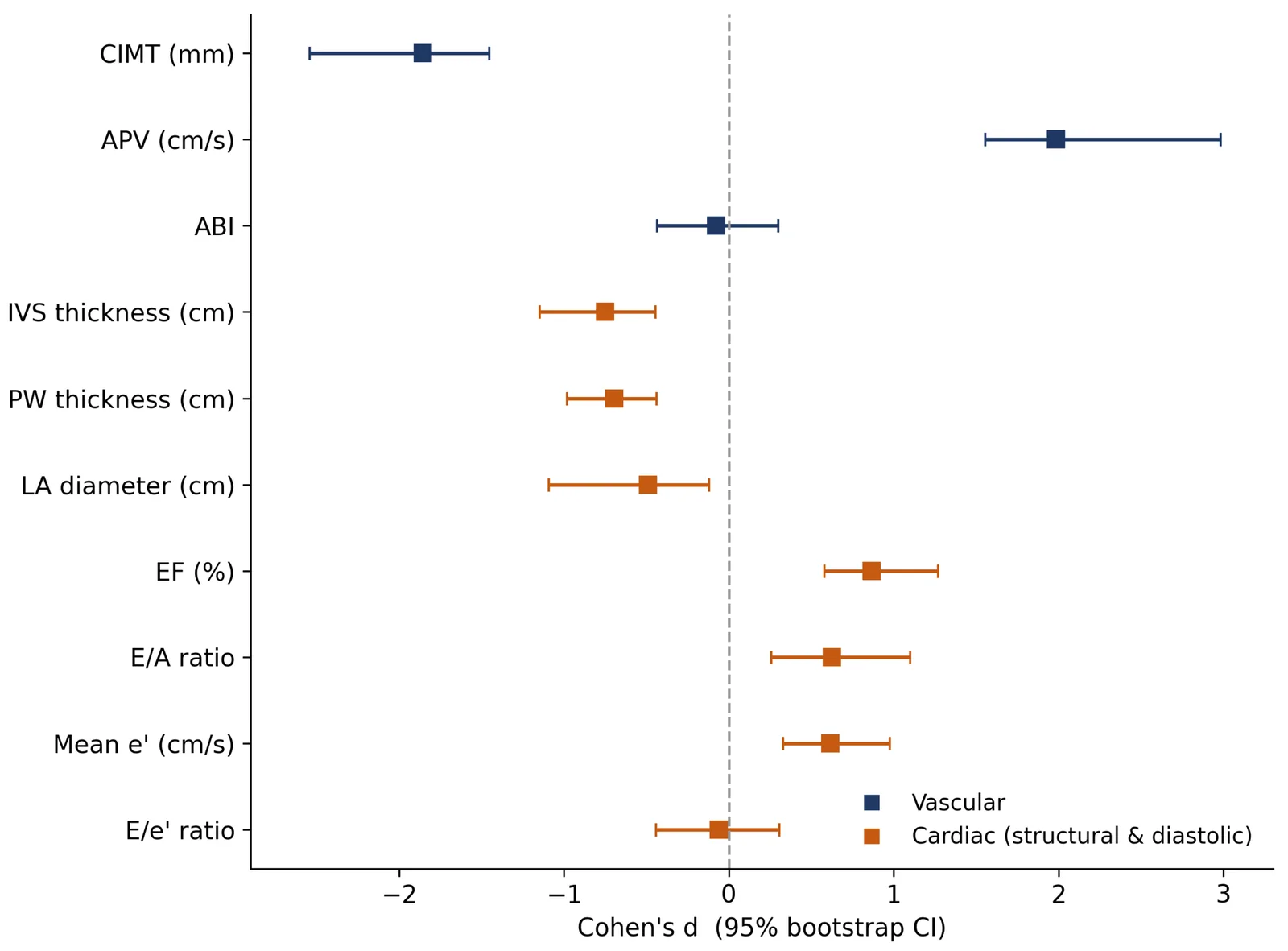
Magnitude and direction of change six months after laparoscopic sleeve gastrectomy. Cohen’s d with 95% bias-corrected and accelerated bootstrap confidence intervals for vascular (CIMT, APV, ABI) and cardiac parameters (IVS, PW, LA, EF, E/A, mean e′, E/e′). Negative values indicate a decrease. The intervals for ABI and E/e′ span zero. Ejection fraction is tabulated in Supplementary Table S2 rather than Table 2. ABI, ankle-brachial index; APV, aortic propagation velocity; CIMT, carotid intima-media thickness; EF, ejection fraction; IVS, interventricular septum; LA, left atrium; PW, posterior wall.

Cardiac indices moved in parallel. IVS thickness fell (d = −0.75), PW thickness fell (d = −0.70) and LA diameter decreased modestly (d = −0.49; all *p* ≤ 0.012). E/A rose (d = 0.63) and mean e′ rose (d = 0.62; both *p* ≤ 0.002). E/e′, an estimate of left ventricular filling pressure, did not change (*p* = 0.871) (Table 2, Figure 1). Against a conservative Bonferroni threshold for the ten paired comparisons (α ≈ 0.005), seven of ten remained significant; ABI, LA diameter and E/e′ did not (Supplementary Note S2).

### 3.4. Type 2 Diabetes and Diastolic Indices

Patients with T2DM (*n* = 18) showed smaller increases in mean e′ and E/A than those without (*n* = 12): Δmean e′ 0.44 ± 1.24 versus 1.71 ± 1.68 cm/s (*t*-test *p* = 0.025; Mann–Whitney U *p* = 0.038) and ΔE/A 0.10 ± 0.30 versus 0.39 ± 0.35 (*p* = 0.024).

**Figure 2:**
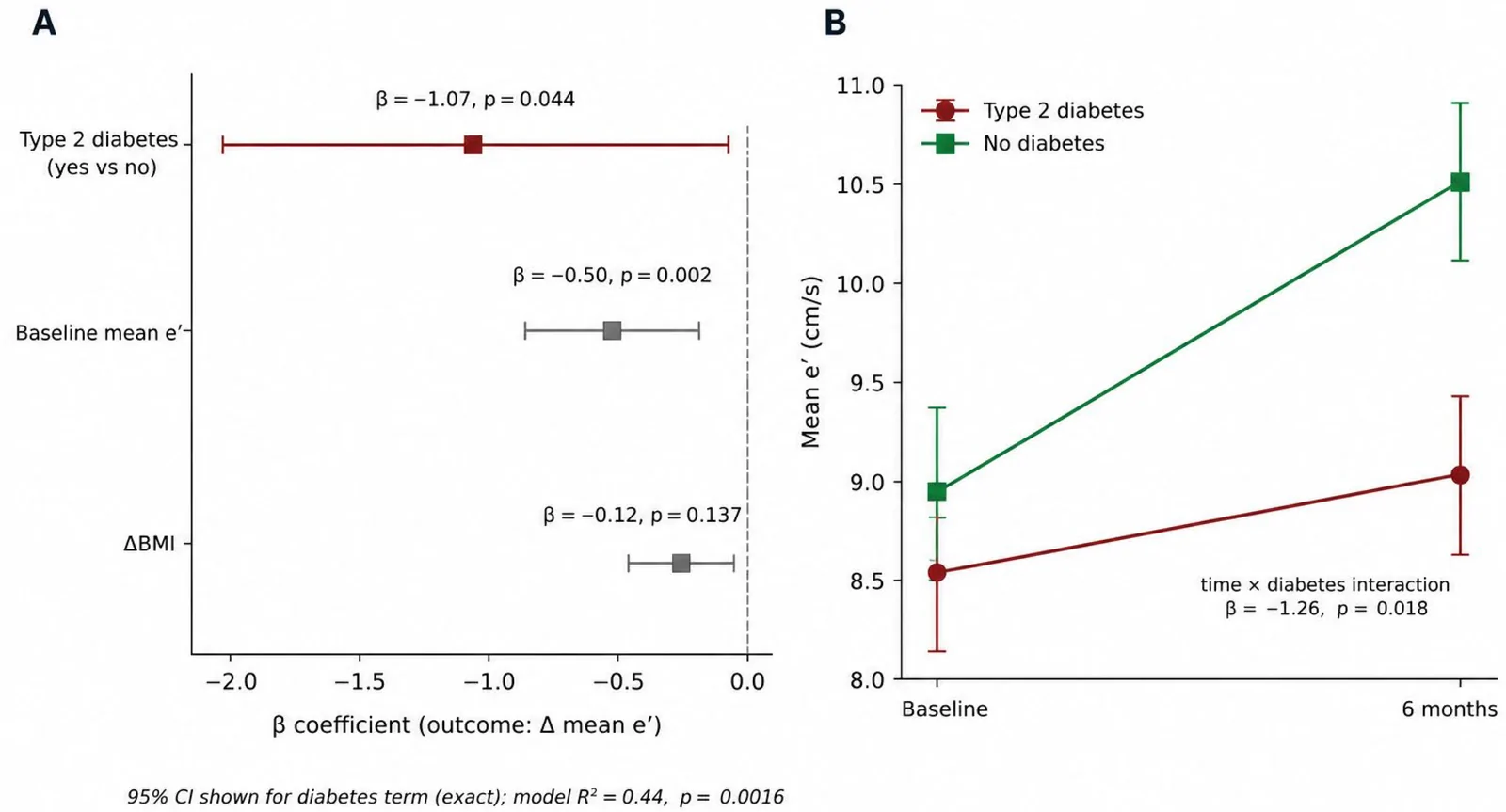
Type 2 diabetes and changes in mean e′. (**A**) Multivariable model of Δmean e′ with type 2 diabetes status, baseline mean e′ and ΔBMI entered simultaneously; the diabetes term remains significant while ΔBMI does not. (**B**) Mean e′ (mean ± SE) at baseline and six months by diabetes status (time × diabetes interaction β = −1.26, *p* = 0.018). BMI, body mass index; SE, standard error.

Weight loss was not equal between the groups: patients with T2DM lost less (ΔBMI −11.86 ± 3.06 versus −14.69 ± 3.01 kg/m^2^, *p* = 0.019), and their baseline HbA1c was higher (8.05 ± 1.80 versus 5.75 ± 0.41%, *p* < 0.001). In the multivariable model, T2DM remained associated with a smaller increase in mean e′ after simultaneous adjustment for baseline mean e′ and ΔBMI (β = −1.07, 95% CI −2.10 to −0.03, *p* = 0.044; model R^2^ = 0.44), while ΔBMI itself was not a significant term (β = −0.12, *p* = 0.137). The association appeared again as a mixed-effects time-by-diabetes interaction (β = −1.26, *p* = 0.018), and repeating the model with E/A gave a concordant result (interaction β = −0.29, *p* = 0.017; Table 3, Figure 2, Supplementary Table S3). No statistically significant difference in vascular changes according to diabetes status was detected, although the study was not powered to establish equivalence between the groups.

The subgroup comparison rests on 18 versus 12 patients and is modestly powered, and the multivariable model carries the overfitting risk described in Section 2.9. We report the finding on the strength of its consistency across three analytical approaches and two diastolic indices rather than on any single *p* value.

### 3.5. Vascular–Diastolic Association (Exploratory)

**Figure 3:**
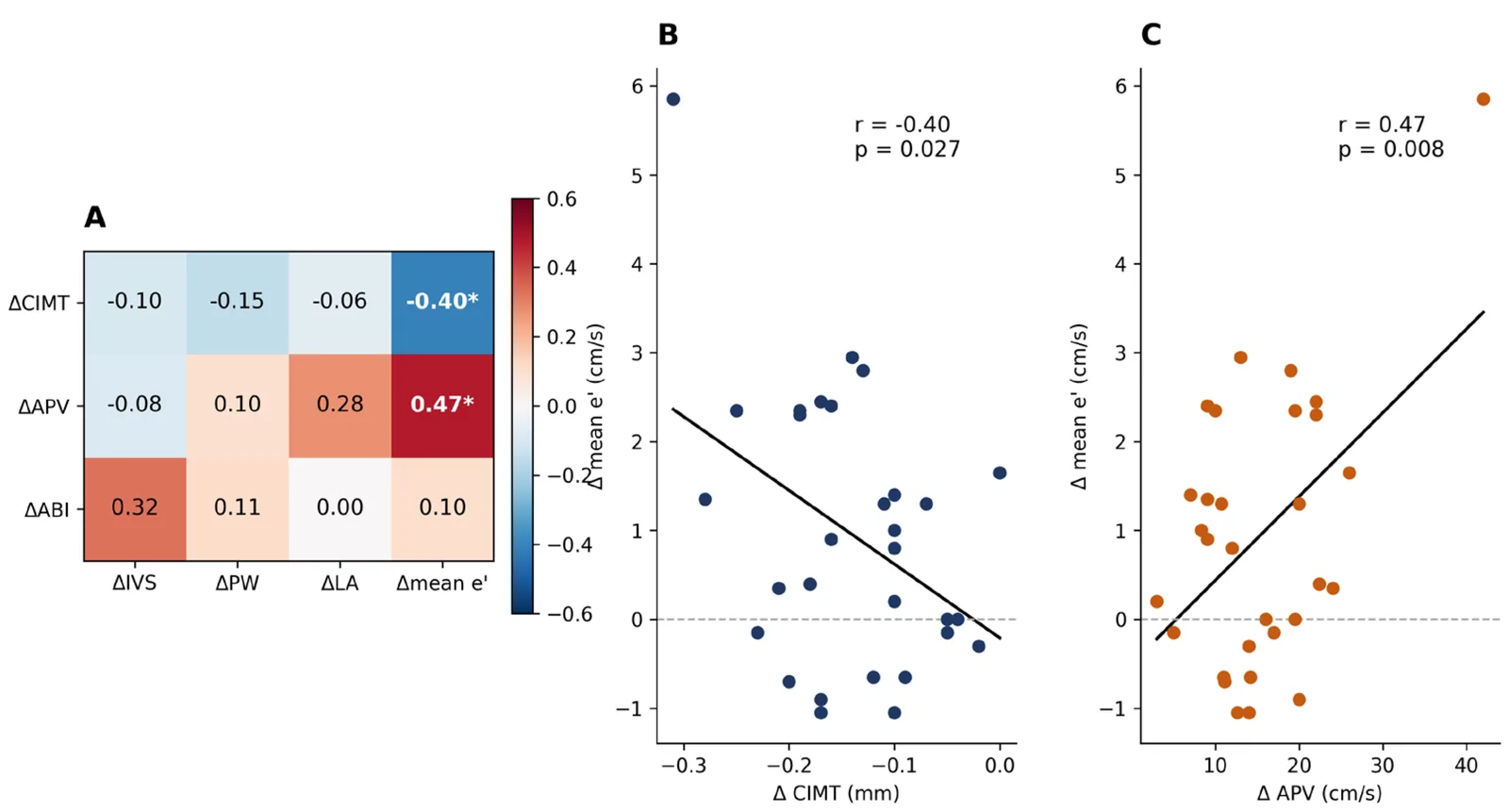
Vascular–cardiac change-score correlations (exploratory). (**A**) Correlation matrix of six-month change scores; cardiac structural markers (IVS, PW, LA) show no association with vascular change, whereas Δmean e′ does. Asterisks mark nominally significant coefficients (*p* < 0.05), uncorrected for multiplicity. (**B**,**C**) Scatter plots of Δmean e′ against ΔCIMT and ΔAPV, with least-squares regression lines. These are exploratory analyses, not study endpoints. Abbreviations as in Figure 1.

Cardiac structural change was unrelated to vascular change: every correlation involving ΔIVS, ΔPW or ΔLA was weak and non-significant (all |r| ≤ 0.32, *p* ≥ 0.087). Diastolic change behaved differently, correlating modestly with both vascular measures (ΔCIMT r = −0.40, *p* = 0.027; ΔAPV r = 0.47, *p* = 0.008) (Figure 3). Only these two correlations were stable in leave-one-out analysis; the structural comparisons were null and stable around zero (Supplementary Table S5). These results are unadjusted for multiplicity and are reported as an exploratory observation, not as a study endpoint.

Change scores were inversely related to baseline values for several outcomes, most strongly for LA diameter (r = −0.79) and IVS thickness (r = −0.68) (Supplementary Table S4). Because a change score contains the baseline value algebraically, such inverse relationships arise from mathematical coupling and cannot be used to quantify regression to the mean; they are reported for transparency only and are interpreted in the Limitations. A legacy SCORE estimate recorded in the original protocol is not reported as an outcome, since that framework has been superseded by SCORE2 and SCORE2-Diabetes [25,26,27,28] and is not validated below 40 years of age; it is retained as a descriptor in Supplementary Table S2.

## 4. Discussion

This study shows that favourable vascular and cardiac changes occur concurrently within six months of laparoscopic sleeve gastrectomy, and that patients with type 2 diabetes show smaller increases in mean e′ and E/A than those without, an association that persists after adjustment for the degree of weight loss. As a secondary, exploratory observation, diastolic—but not structural—cardiac change tracked vascular change.

### 4.1. Comparison with Previous Literature

The vascular findings are consistent in direction and magnitude with earlier surgical weight-loss cohorts reporting CIMT regression and improved aortic propagation [17,18,19]. The ankle-brachial index, by contrast, showed no measurable change over the same interval.

The cardiac findings sit within a substantial literature on reverse remodelling after bariatric surgery: regression of wall thickness, modest atrial change and improved diastolic filling have been reported repeatedly in imaging series and meta-analyses [16,29,30,31,32,33,34,35], including a 2026 study in metabolically healthy obese patients that found a comparable rise in E/A at six months against a non-surgical control group [36].

Two recent observations bear directly on our results. A 2025 dose–response analysis found that cardiac reverse remodelling correlated only weakly with the magnitude of BMI reduction [14], which is consistent with our own model, in which the magnitude of BMI reduction was not independently associated with the change in mean e′ in this cohort. More pointedly, a 2025 speckle-tracking study of 58 patients found that those with obesity and diabetes had lower global longitudinal strain at every timepoint and a smaller six-month gain than those with obesity alone [15]. That study used a different index, a different technique and a different population, and reached the same conclusion as ours. Two independent cohorts converging on the same asymmetry is a stronger basis for the observation than either alone.

### 4.2. Potential Mechanisms

If diabetic myocardial disease involves interstitial fibrosis and microvascular dysfunction rather than load alone [20,21], then relieving haemodynamic load and adiposity would be expected to restore relaxation only partially. The vascular measures that changed in this cohort—carotid intima-media thickness and aortic propagation velocity—depend more directly on metabolic and haemodynamic status, and no statistically significant difference in their change according to diabetes status was detected. This is consistent with quantitative perfusion cardiac magnetic resonance data showing that, although coronary microvascular function improves after bariatric surgery, the increase in stress myocardial blood flow is smaller in patients with preoperative type 2 diabetes [37]. Our data cannot address microvascular function directly, but the parallel between that observation and the smaller increase in mean e′ seen here is notable.

The dissociation between cardiac structural and functional change is harder to interpret and we do not attempt a mechanistic account. It may reflect different time courses, or different determinants of chamber geometry versus myocardial relaxation. Testing this would require tissue characterisation or strain imaging, neither of which was available here.

### 4.3. Clinical Implications

If confirmed in larger studies, these observations would suggest that postoperative changes in diastolic indices may not be uniform across patients. Whether patients with T2DM would benefit from closer or more prolonged echocardiographic follow-up after surgery is a question for future research rather than a clinical recommendation supported by this small exploratory study. Nothing in these data argues against surgery in diabetes—that group improved metabolically, vascularly and, to a lesser degree, diastolically—only against assuming that improvement in myocardial indices follows automatically from weight loss. This sits alongside two established observations: bariatric surgery reduces long-term cardiovascular events [38,39], so the direction of benefit is not in question; and cardiovascular risk in type 2 diabetes persists despite effective glucose-lowering therapy [40], so smaller myocardial changes after metabolically successful surgery would not be anomalous.

### 4.4. Future Research

The obvious next step is a larger cohort with a non-surgical comparator, strain imaging or tissue characterisation, prospectively recorded medication data, and follow-up beyond six months, powered to test the diabetes interaction as a primary endpoint rather than to detect it. Whether diabetes duration, glycaemic control or the presence of microvascular complications predicts the degree of attenuation is a question our sample cannot answer and a natural target for such a study.

### 4.5. Limitations

Attrition is the principal limitation. Twenty of the 50 enrolled patients (40.0%) were not reassessed during the COVID-19 pandemic, when elective outpatient follow-up was substantially disrupted. Individual reasons for loss to follow-up were not systematically recorded. Importantly, completers were younger, less frequently hyperlipidaemic and had a slightly higher baseline ejection fraction than non-completers. Selection bias therefore cannot be excluded, and the results may not generalise to older patients with a greater atherogenic burden. No statistically significant difference was detected in the vascular or echocardiographic variables, in adiposity, or in the prevalence of type 2 diabetes; the comparison was descriptive and was not powered to establish equivalence, so it does not exclude differences in these variables. The analysed sample of 30 further constrains precision throughout.

There was no non-surgical comparator group. Secular change, regression to the mean and concurrent medical therapy therefore cannot be formally separated from the effect of surgery. Change scores were inversely related to baseline values for several outcomes (Supplementary Table S4). We do not treat these correlations as a measure of regression to the mean: because the change score contains the baseline value algebraically, an inverse relationship of this kind is expected whenever measurement variability is present, and its magnitude is determined by the test–retest correlation and the ratio of variances rather than by the size of any regression effect. The observed relationships are therefore compatible with regression to the mean, with genuine baseline-dependent change, or with both, and the relative contributions cannot be separated without repeated baseline measurements or a non-surgical comparator. We make no causal claim.

Detailed medication class and dose data were not systematically recorded for all participants at both timepoints; longitudinal changes in medical therapy could therefore not be reliably characterised. This is a material limitation. Antihypertensive and glucose-lowering agents influence loading conditions, haemodynamics and metabolic status, all of which bear on the echocardiographic and vascular measurements reported here. Unrecorded changes in treatment are therefore an uncontrolled confounder of these findings, and the changes we observed should be attributed to the post-operative state as a whole rather than to weight loss in isolation.

The diabetes subgroup comparison (18 versus 12) is modestly powered, and diabetes duration and post-operative changes in glucose-lowering therapy were not available as covariates; baseline HbA1c was higher in the T2DM group, as expected, and could not be separated from diabetes status itself. The multivariable model carries three predictors on 30 observations, a ratio at the lower end of the usually recommended 10–15 observations per variable, so its coefficients may be unstable and subject to overfitting.

Follow-up was six months, beyond which weight and cardiometabolic trajectories continue to evolve [41]. All measurements were operator-dependent; a single experienced operator, blinded to diabetes status and laboratory results but not to patient identity or study timepoint, performed every study on the same equipment, which limits inter-observer variability, but intra-observer reproducibility was not formally quantified. Left atrial volume index and tricuspid regurgitant velocity were not recorded, precluding formal grading of diastolic dysfunction, and no strain imaging was performed; the diastolic findings therefore describe change in continuous indices and cannot be translated into diastolic function grades. The number of patients excluded under each individual exclusion criterion was not recorded. Finally, the exploratory vascular–diastolic correlations were not corrected for multiple comparisons.

## 5. Conclusions

Carotid intima-media thickness and aortic propagation velocity improved within six months of laparoscopic sleeve gastrectomy, alongside favourable changes in several cardiac structural and diastolic indices, while the ankle-brachial index and E/e′ remained unchanged. Type 2 diabetes was associated with a smaller increase in mean e′, and this association persisted after adjustment for the degree of weight loss, which was itself lower in the diabetic group. In a cohort of this size, with 40% pandemic-related attrition, uncontrolled changes in medication and no non-surgical control group, regression to the mean cannot be separated from the postoperative changes observed and no causal inference is warranted. These observations are hypothesis-generating and require confirmation in larger, controlled studies before any implication for postoperative follow-up can be considered.

## Figures and Tables

**Table 1:** Clinical, haemodynamic and biochemical characteristics before and six months after laparoscopic sleeve gastrectomy (*n* = 30).

Variable	Baseline	6 Months	*p*
BMI (kg/m^2^)	45.5 ± 6.6	32.5 ± 5.8	<0.001
Systolic BP (mmHg)	150.2 ± 16.7	117.5 ± 13.2	<0.001
Diastolic BP (mmHg)	79.2 ± 8.9	67.8 ± 7.6	<0.001
Hypertension at baseline, n (%)	21 (70.0)	— a	—
Type 2 diabetes at baseline, n (%)	18 (60.0)	— a	—
Hyperlipidaemia at baseline, n (%)	11 (36.7)	— a	—
Current smoking, n (%)	14 (46.7)	5 (16.7)	0.004
Fasting glucose (mg/dL)	159.7 ± 78.9	97.9 ± 24.2	<0.001
HbA1c (%)	7.1 ± 1.8	5.6 ± 0.9	<0.001
Total cholesterol (mg/dL)	210.8 ± 40.1	192.1 ± 48.1	0.030
LDL-C (mg/dL)	141.6 ± 34.2	123.0 ± 38.3	0.002
HDL-C (mg/dL)	49.5 ± 11.1	52.6 ± 12.3	0.044
Triglycerides (mg/dL)	205.6 ± 138.8	129.4 ± 69.3	0.002

Values are mean ± SD or n (%). *p* values from the paired *t*-test or, for smoking, the McNemar test. a Hypertension, type 2 diabetes and hyperlipidaemia were defined by an established diagnosis or the use of the corresponding therapy; no prespecified remission criteria were applied and medication was not systematically recorded at both timepoints, so six-month status is not reported for these variables. BMI, body mass index; BP, blood pressure; HbA1c, glycated haemoglobin; HDL-C, high-density lipoprotein cholesterol; LDL-C, low-density lipoprotein cholesterol.

**Table 2:** Vascular and cardiac outcomes at six months (*n* = 30).

Parameter	Baseline	6 Months	*p*	Cohen’s d
Vascular				
CIMT (mm)	0.80 ± 0.12	0.66 ± 0.11	<0.001	−1.86
APV (cm/s)	52.0 ± 10.1	67.4 ± 10.8	<0.001	1.99
ABI	1.07 ± 0.21	1.06 ± 0.07	0.679	−0.08
Cardiac				
IVS thickness (cm)	1.13 ± 0.13	1.05 ± 0.10	<0.001	−0.75
PW thickness (cm)	1.03 ± 0.05	1.00 ± 0.05	0.001	−0.70
LA diameter (cm)	4.24 ± 0.47	4.12 ± 0.31	0.012	−0.49
E/A ratio	1.01 ± 0.31	1.23 ± 0.36	0.002	0.63
Mean e′ (cm/s)	8.67 ± 1.57	9.62 ± 1.65	0.002	0.62
E/e′ ratio	8.53 ± 2.54	8.39 ± 1.43	0.871	−0.06

Values are mean ± SD. *p* values from the paired *t*-test, except E/e′ (Wilcoxon signed-rank). Ejection fraction and legacy SCORE are in Supplementary Table S2. ABI, ankle-brachial index; APV, aortic propagation velocity; CIMT, carotid intima-media thickness; IVS, interventricular septum; LA, left atrium; PW, posterior wall.

**Table 3:** Type 2 diabetes and changes in diastolic indices.

Analysis	Estimate	*p*
Δmean e′: T2DM vs. no T2DM	0.44 ± 1.24 vs. 1.71 ± 1.68 cm/s	0.025
ΔE/A: T2DM vs. no T2DM	0.10 ± 0.30 vs. 0.39 ± 0.35	0.024
ΔBMI: T2DM vs. no T2DM	−11.86 ± 3.06 vs. −14.69 ± 3.01 kg/m^2^	0.019
Multivariable β for T2DM (adjusted for baseline mean e′, ΔBMI)	−1.07 (95% CI −2.10 to −0.03)	0.044
ΔBMI term in the same model	β = −0.12 (95% CI −0.27 to 0.04)	0.137
Mixed-effects time × T2DM interaction (mean e′)	β = −1.26	0.018
Mixed-effects time × T2DM interaction (E/A)	β = −0.29	0.017

Full subgroup statistics in Supplementary Table S3. BMI, body mass index; CI, confidence interval; T2DM, type 2 diabetes mellitus.

## Data Availability

The de-identified dataset and analysis code supporting the findings of this study are available from the corresponding author upon reasonable request. The data are not publicly available because they contain information that could compromise the privacy of research participants.

## Supplementary Materials

The following supporting information can be downloaded at: https://www.mdpi.com/article/10.3390/medicina62101896/s1, Supplementary Methods; Table S1: Extended clinical characteristics; Table S2: Ejection fraction and legacy SCORE; Table S3: Diabetes subgroup statistics and the parallel E/A model; Table S4: Baseline-versus-change analysis; Table S5: Full vascular–cardiac correlation matrix with leave-one-out sensitivity range; Table S6: Completers versus non-completers at baseline; Statistical Notes S1–S4.
