# Supplementary material for: Effects of Laparoscopic Sleeve Gastrectomy on Vascular and Myocardial Recovery: Type 2 Diabetes and Echocardiographic Indices of Diastolic Function
Source: Medicina (Kaunas). 2026 Sep 30;62(10):1896. doi: 10.3390/medicina62101896 (PMC13707178; doi:10.3390/medicina62101896)
Supplement: Supplementary file 1 [file medicina-62-01896-s001.zip › medicina-4521265-supplementary.pdf]

Supplementary Materials

Effects of Laparoscopic Sleeve Gastrectomy on Vascular and Myocardial Recovery: Type 2 Diabetes and Echocardiographic Indices of Diastolic Function

Samet Yılmaz, Kayıhan Karaman, Zeki Özsoy and Ataç Çelik

This file contains the Supplementary Methods, Tables S1–S6 and Statistical Notes S1–S4 referred to in the main manuscript. All supporting analyses are presented in tabular form; there are no supplementary figures.

Supplementary Methods

Fifty consecutive patients with severe obesity scheduled for laparoscopic sleeve gastrectomy were enrolled and underwent baseline assessment. Twenty were not reassessed during pandemic-related disruption of elective outpatient follow-up; individual reasons for loss to follow-up were not systematically recorded. The analysis population comprised the 30 patients with complete paired baseline and six-month data. Within this analysis population there were no post-enrolment exclusions and no missing values for any primary variable at either timepoint, so no imputation was required. Baseline characteristics of completers and non-completers are compared in Table S6.

Before analysis the research database was independently re-extracted from the primary study record and every primary variable was recomputed from patient-level values. Cohen's d for paired comparisons was the mean within-patient difference divided by its standard deviation, with 95% confidence intervals from 5000 bias-corrected and accelerated bootstrap resamples of the paired differences (fixed random seed; Python 3, SciPy). Effect sizes are reported for every paired outcome, including the ankle-brachial index.

The multivariable model for Δmean e′ included type 2 diabetes status, baseline mean e′ and ΔBMI, entered simultaneously with no stepwise selection. The mixed-effects model treated mean e′ (and, in a parallel model, E/A) as the longitudinal outcome across two timepoints, with fixed effects for time, diabetes status and their interaction, and a random intercept per patient. Completers and non-completers were compared with Welch's independent-samples t-test for continuous variables and Fisher's exact test for categorical variables.

For every vascular–cardiac change-score correlation, a leave-one-out analysis recalculated the Pearson coefficient after sequential exclusion of each of the 30 patients. A correlation was considered robust if this range did not cross zero.

Table S1. Extended clinical characteristics (n = 30).

Variable	Baseline	6 months	p
Weight (kg)	129.6 ± 21.0	92.5 ± 16.9	<0.001
Total cholesterol (mg/dL)	210.8 ± 40.1	192.1 ± 48.1	0.030
HDL-C (mg/dL)	49.5 ± 11.1	52.6 ± 12.3	0.044
Triglycerides (mg/dL)	205.6 ± 138.8	129.4 ± 69.3	0.002
Creatinine (mg/dL)	0.79 ± 0.19	0.77 ± 0.16	0.329

Complements Table 1 of the main manuscript, in which blood pressure, glycaemic and lipid variables are now reported. HDL-C, high-density lipoprotein cholesterol.

Table S2. Ejection fraction and legacy SCORE (exploratory).

Parameter	Baseline	6 months	p	Note
EF (%)	63.2 ± 2.6	64.9 ± 2.3	<0.001	Within the normal range throughout; a safety marker, not a remodelling endpoint (d = 0.87)
SCORE (legacy)	1 (1–3)	1 (0–1)	<0.001	Superseded by SCORE2 / SCORE2-Diabetes; not validated below 40 years

SCORE is presented only as a legacy descriptor. No inferential analysis involving SCORE appears in the main manuscript. EF, ejection fraction.

Table S3. Diabetes subgroup statistics and the parallel E/A model.

Analysis	Estimate	Method	p
Δmean e′: T2DM vs no T2DM	0.44 vs 1.71 cm/s	t-test	0.025
Δmean e′: T2DM vs no T2DM	0.44 vs 1.71 cm/s	Mann–Whitney U	0.038
ΔE/A: T2DM vs no T2DM	0.10 vs 0.39	t-test	0.024
ΔE/A: T2DM vs no T2DM	0.10 vs 0.39	Mann–Whitney U	0.049
ΔBMI: T2DM vs no T2DM	−11.86 vs −14.69 kg/m²	t-test	0.019
Baseline HbA1c: T2DM vs no T2DM	8.05 vs 5.75 %	t-test	<0.001
Multivariable model fit (Δmean e′)	R^2 = 0.440, adj. R^2 = 0.375	F-test	0.0016
T2DM term	β = −1.07 (−2.10 to −0.03)	—	0.044
Baseline mean e′ term	β = −0.50 (−0.80 to −0.20)	—	0.002
ΔBMI term	β = −0.12 (−0.27 to 0.04)	—	0.137
Mixed-effects interaction (mean e′)	β = −1.26	—	0.018
Mixed-effects interaction (E/A)	β = −0.29	—	0.017

The multivariable model estimates three predictors on 30 observations; coefficients should be interpreted in the light of this sample-to-variable ratio (Note S2). BMI, body mass index; HbA1c, glycated haemoglobin; T2DM, type 2 diabetes mellitus.

Table S4. Baseline value versus subsequent change.

Parameter	r (baseline vs Δ)	p
Left atrial diameter	−0.79	<0.001
Interventricular septal thickness	−0.68	<0.001
Mean e′	−0.44	0.016
E/A ratio	−0.40	0.031

Reported for transparency in response to a reviewer request. These coefficients must not be read as a measure of regression to the mean. Because the change score contains the baseline

value algebraically, an inverse baseline–change correlation is expected whenever measurement variability is present, and its magnitude is fixed by the test–retest correlation and the ratio of variances rather than by the size of any regression effect. The observed relationships are compatible with regression to the mean, with genuine baseline-dependent recovery, or with both; the contributions cannot be separated without repeated baseline measurements or a non-surgical comparator.

Table S5. Vascular–cardiac change-score correlations with leave-one-out sensitivity range.

Comparison	r	p	Leave-one-out range
ΔCIMT vs ΔIVS	−0.10	0.615	−0.15 to 0.05
ΔCIMT vs ΔPW	−0.15	0.430	−0.27 to −0.08
ΔCIMT vs ΔLA	−0.06	0.739	−0.15 to 0.01
ΔCIMT vs Δmean e′	−0.40	0.027	−0.47 to −0.21
ΔAPV vs ΔIVS	−0.08	0.669	−0.16 to 0.02
ΔAPV vs ΔPW	0.10	0.609	0.02 to 0.16
ΔAPV vs ΔLA	0.28	0.141	0.22 to 0.34
ΔAPV vs Δmean e′	0.47	0.008	0.14 to 0.52
ΔABI vs ΔIVS	0.32	0.087	0.26 to 0.40
ΔABI vs ΔPW	0.11	0.564	0.06 to 0.19
ΔABI vs ΔLA	0.00	0.985	−0.05 to 0.07
ΔABI vs Δmean e′	0.10	0.603	0.03 to 0.18

Underlies Figure 3 of the main manuscript. Only ΔCIMT and ΔAPV (both versus Δmean e') reached nominal significance and remained leave-one-out stable; all structural comparisons were null and stable around zero. Coefficients are reported to two decimal places throughout and match Figure 3 exactly.

Table S6. Baseline characteristics of patients who completed and did not complete six-month follow-up.

Variable	Completers (n = 30)	Non-completers (n = 20)	p

Age (years)	43.8 ± 10.6	52.4 ± 5.8	0.001
Female sex, n (%)	17 (56.7)	12 (60.0)	1.000
BMI (kg/m²)	45.5 ± 6.6	42.6 ± 3.9	0.057
Systolic BP (mmHg)	150.2 ± 16.7	143.2 ± 17.5	0.165
Diastolic BP (mmHg)	79.2 ± 8.9	80.5 ± 8.0	0.585
Hypertension, n (%)	21 (70.0)	15 (75.0)	0.758
Type 2 diabetes, n (%)	18 (60.0)	13 (65.0)	0.774
Hyperlipidaemia, n (%)	11 (36.7)	15 (75.0)	0.010
Current smoking, n (%)	14 (46.7)	10 (50.0)	1.000
HbA1c (%)	7.13 ± 1.81	7.61 ± 1.54	0.326
Fasting glucose (mg/dL)	159.7 ± 78.9	148.4 ± 54.1	0.550
CIMT (mm)	0.80 ± 0.12	0.83 ± 0.10	0.341
APV (cm/s)	52.0 ± 10.1	54.3 ± 10.4	0.448
ABI	1.07 ± 0.21	0.96 ± 0.19	0.069
IVS thickness (cm)	1.13 ± 0.13	1.17 ± 0.12	0.239
PW thickness (cm)	1.03 ± 0.05	1.07 ± 0.10	0.069
LA diameter (cm)	4.24 ± 0.47	4.28 ± 0.41	0.752
LVEF (%)	63.2 ± 2.6	60.5 ± 3.5	0.006
E/A ratio	1.01 ± 0.31	1.07 ± 0.31	0.468
Mean e′ (cm/s)	8.67 ± 1.57	8.64 ± 0.88	0.935
E/e′ ratio	8.53 ± 2.54	8.51 ± 1.42	0.967

Values are mean ± SD or n (%). p values from Welch's independent-samples t-test or Fisher's exact test. Completers were younger, less often hyperlipidaemic and had a slightly higher baseline ejection fraction. No statistically significant difference was detected in the remaining variables; this descriptive comparison involved 21 variables without correction for multiplicity and was not powered to establish equivalence, and several comparisons were borderline (BMI, ABI, posterior wall thickness). ABI, ankle-brachial index; APV, aortic propagation velocity; BMI, body mass index; BP, blood pressure; CIMT, carotid intima-media thickness; HbA1c, glycated haemoglobin; IVS, interventricular septum; LA, left atrium; LVEF, left ventricular ejection fraction; PW, posterior wall.

Statistical Notes

Note S1 — Ankle-brachial index. ABI was re-analysed from paired patient-level Doppler pressures at both timepoints. The paired effect size is $d = -0.08$ (95% CI −0.43 to 0.30, $p = 0.679$, Wilcoxon $p = 0.773$): mean ABI did not change over six months. This supersedes the

summary reported in the original submission. No categorical or threshold-based analysis of ABI was performed.

Note S2 — Multiplicity and model stability. Ten paired vascular and cardiac comparisons were performed. Against a Bonferroni-adjusted threshold of $\alpha \approx 0.005$, seven of ten remain significant; ABI, left atrial diameter and E/e′ do not. The multivariable model estimates three predictors on 30 observations, a ratio of ten observations per variable, at the lower end of the usually recommended 10–15 observations per variable; coefficients may therefore be unstable and the model at risk of overfitting. The mixed-effects model is reported alongside as a complementary longitudinal analysis, but uses repeated measurements on the same 30 participants and is therefore subject to the same constraint on independent observations. The correlation matrix (Table S5) is exploratory and reported uncorrected, with all values shown. The baseline-versus-change coefficients in Table S4 are descriptive only and are not interpreted as effect estimates (see the note to that table).

Note S3 — Attrition and missing data. Of the 50 patients enrolled, 20 were not reassessed during pandemic-related disruption of elective outpatient follow-up, as described in Section 2.1 of the main manuscript; individual reasons were not systematically recorded. Baseline characteristics of completers and non-completers are compared in Table S6: completers were younger, less often hyperlipidaemic and had a slightly higher baseline ejection fraction. No statistically significant difference was detected in any vascular or echocardiographic variable, in adiposity, or in the prevalence of type 2 diabetes, but the comparison was descriptive, involved 21 variables without correction for multiplicity, and was not powered to establish equivalence; several comparisons were borderline (BMI $p = 0.057$, ABI $p = 0.069$, posterior wall thickness $p = 0.069$). Selection bias therefore cannot be excluded, and the results may not generalise to older patients with a greater

atherogenic burden. Within the 30-patient analysis population there were no missing values for any primary variable at either timepoint and no imputation was required.

Note S4 — Reproducibility. Analyses were performed using IBM SPSS Statistics version 18.0 (IBM Corp., Armonk, NY, USA), with Python 3 (SciPy, statsmodels) additionally used during manuscript preparation for selected supplementary calculations and verification; bootstrap resampling used 5000 resamples with the BCa method and a fixed seed. Aortic propagation velocity is reported in cm/s. Code and the de-identified dataset are available from the corresponding author upon reasonable request.
